# Reply to Twycross et al. Comment on “Ramos-Rincon et al. Palliative Sedation in COVID-19 End-of-Life Care. Retrospective Cohort Study. *Medicina* 2021, *57*, 873”

**DOI:** 10.3390/medicina58010083

**Published:** 2022-01-06

**Authors:** Jose-Manuel Ramos-Rincon, Manuel Priego-Valladares

**Affiliations:** 1 Internal Medicine Department, Alicante General University Hospital—Alicante Institute of Health and Biomedical Research (ISABIAL), 03010 Alicante, Spain; 2 Clinical Medicine Department, Miguel Hernández University, 03550 Elche, Spain; 3 Palliative Care Unit and Internal Medicine Department, Alicante General University Hospital—Alicante Institute of Health and Biomedical Research (ISABIAL), 03010 Alicante, Spain; priego_man@gva.es

First of all, we want to thank Twycross, Wong, and Vivat [1] for their interest in our study [2]. We agree with the considerations you have made; however, we would like to clarify a few points.

The SARS-CoV-2 pandemic has greatly challenged health systems, forcing professionals to face an unknown disease, with high hospital mortality (20%) and rapid progression [3]. Healthcare demands have surpassed the supply of both human and material resources, leading to shortages of even the most basic medication for symptom control [4]. In this context, professionals from different specialties, without deep knowledge in palliative care, had to care for patients with COVID-19 who did not respond to available treatments, could not access intensive care units (ICU), and presented high levels of suffering from unrelieved symptoms. Decision-making in this situation was highly complex, as patients were frequently unable to provide informed consent for the administration of palliative sedation, and, moreover, they were isolated from their families. In almost all cases in which palliative sedation was called for, continuous deep sedation was necessary due to the severity and rapid evolution of the symptoms. In our retrospective study carried out by professionals with no previous experience in palliative care, the level of consciousness of the patients was not monitored.

In all our included patients receiving palliative sedation, the intention of the care team, as documented in the clinical history, was to decrease the level of consciousness due to the presence of refractory symptoms. The initial doses used were relatively low (0.6–1 mg/h of midazolam), mainly because patients were of advanced age (>80 years on average) and, in many cases, presented (acute or chronic) renal failure and/or liver failure. The drugs used in palliative sedation were midazolam and levomepromazine in all cases, except in four that may not be considered palliative sedation. Although some studies consider haloperidol as palliative sedation [5], this drug produces little sedation, and high doses may produce side effects that limit its use. The rest of the drugs described in Table 2 of our article (morphine, hyoscine butyl bromide, and haloperidol [2]) were co-administered to control symptoms.

The fact that the survival time after the start of palliative sedation is short (<24 h) indicates that the evolution of the disease was very rapid and palliative sedation was prescribed very late. However, survival was similar between deceased patients who required palliative sedation and those who did not (13 days vs. 12 days), indicating that palliative sedation did not accelerate death.

In a recent study that took place in Spanish hospitals [6] on palliative sedation during the pandemic, the rate of palliative sedation is somewhat higher than in ours (57% vs. 44% of deceased patients). Midazolam was also the most widely used drug, but mean daily doses were higher than those collected in our study (43 mg vs. 15 mg). However, the doses we report were initial doses that were later adjusted according to the patients’ needs.

The use of palliative sedation does not necessarily indicate that the palliative care is of high quality. Palliative sedation must comply with ethical safeguards with regard to decision-making, informed consent, and the proportionate application of the procedure. However, failing to administer palliative sedation in a patient with refractory suffering produced by a terminal illness in the last hours or days of life should be considered medical malpractice. In our acute care hospitals, professionals with little to no training in palliative care are often reluctant to prescribe drugs such as morphine or midazolam for the symptomatic control of patients at the end of life due to the false assumption that they could hasten or cause death.

For all these reasons, we consider that, regardless of the debate on the appropriate use of terminology, our work provides a useful insight into what happened in a tertiary Spanish university hospital in patients who died from COVID-19 during the most difficult phases of the pandemic, taking into account the insufficient availability of palliative care teams in our environment.
